# *Plasmodium knowlesi* Skeleton-Binding Protein 1 Localizes to the ‘Sinton and Mulligan’ Stipplings in the Cytoplasm of Monkey and Human Erythrocytes

**DOI:** 10.1371/journal.pone.0164272

**Published:** 2016-10-12

**Authors:** Amuza Byaruhanga Lucky, Miako Sakaguchi, Yuko Katakai, Satoru Kawai, Kazuhide Yahata, Thomas J. Templeton, Osamu Kaneko

**Affiliations:** 1 Department of Protozoology, Institute of Tropical Medicine (NEKKEN), Nagasaki University, Sakamoto, Nagasaki 852-8523, Japan; 2 Leading program, Graduate School of Biomedical Sciences, Nagasaki University, Sakamoto, Nagasaki 852-8523, Japan; 3 Central Laboratory, Institute of Tropical Medicine (NEKKEN), Nagasaki University, Sakamoto, Nagasaki 852-8523, Japan; 4 The Corporation for Production and Research of Laboratory Primates, Tsukuba 305-0843, Japan; 5 Department of Tropical Medicine and Parasitology, Dokkyo Medical University, Tochigi 321-0293, Japan; 6 Department of Microbiology and Immunology, Weill Cornell Medical College, New York 10021, United States of America; Institut national de la santé et de la recherche médicale - Institut Cochin, FRANCE

## Abstract

The malaria parasite, *Plasmodium*, exports protein products to the infected erythrocyte to introduce modifications necessary for the establishment of nutrient acquisition and surface display of host interaction ligands. Erythrocyte remodeling impacts parasite virulence and disease pathology and is well documented for the human malaria parasite *Plasmodium falciparum*, but has been less described for other *Plasmodium* species. For *P*. *falciparum*, the exported protein skeleton-binding protein 1 (PfSBP1) is involved in the trafficking of erythrocyte surface ligands and localized to membranous structures within the infected erythrocyte, termed Maurer's clefts. In this study, we analyzed SBP1 orthologs across the *Plasmodium* genus by BLAST analysis and conserved gene synteny, which were also recently described by de Niz *et al*. (2016). To evaluate the localization of an SBP1 ortholog, we utilized the zoonotic malaria parasite, *Plasmodium knowlesi*. Immunofluorescence assay of transgenic *P*. *knowlesi* parasites expressing epitope-tagged recombinant PkSBP1 revealed a punctate staining pattern reminiscent of Maurer's clefts, following infection of either monkey or human erythrocytes. The recombinant PkSBP1-positive puncta co-localized with Giemsa-stained structures, known as ‘Sinton and Mulligan’ stipplings. Immunoelectron microscopy also showed that recombinant PkSBP1 localizes within or on the membranous structures akin to the Maurer's clefts. The recombinant PkSBP1 expressed in *P*. *falciparum*-infected erythrocytes co-localized with PfSBP1 at the Maurer's clefts, indicating an analogous trafficking pattern. A member of the *P*. *knowlesi* 2TM protein family was also expressed and localized to membranous structures in infected monkey erythrocytes. These results suggest that the trafficking machinery and induced erythrocyte cellular structures of *P*. *knowlesi* are similar following infection of both monkey and human erythrocytes, and are conserved with *P*. *falciparum*.

## Introduction

Despite a 37% decrease in the global incidence of malaria due to interventions in the past 15 years, the malaria parasite has consistently innovated ways to thwart control efforts. Resistance to first-line drugs for treating malaria has long been a challenge and now antimalarial drugs must be developed to limit the emergence of artemisinin-resistant parasites [1]. Control efforts largely focus on the virulent *Plasmodium falciparum*, and the widespread *Plasmodium vivax*, with lesser emphasis targeting other *Plasmodium* species that cause human malaria. The World Health Organization has recognized *Plasmodium knowlesi* as the causative agent of the fifth human malaria [2]. *P*. *knowlesi* infection sometimes results in fatal illness due to high levels of parasitemia as a consequence of the short 24-hour blood stage replication cycle of the parasite [3]. *P*. *knowlesi* is traditionally considered a non-human malaria parasite, found in Southeast Asia infecting macaque monkeys, and humans as a zoonotic infection [4]. For such zoonotic parasites, increased direct or close contact with humans and animals results from urbanization, and for *P*. *knowlesi* represents another obstacle in the general fight against malaria. Human-animal contact is pivotal in expanding host niches potentiating the adaptation of simian *Plasmodium* species to human hosts [5,6].

The increased clinical descriptions of naturally acquired human infection [7,8] underscore the importance of *P*. *knowlesi* in malaria research, with the complementing view that it also provides an experimental model for *P*. *vivax*. Before it became a public health concern, *P*. *knowlesi* had been an invaluable tool in *in vivo* and *in vitro* studies of malaria parasite processes such as antigenic variation [9] and host invasion [10]. Lines of *P*. *knowlesi* have been recently adapted to *in vitro* culture using human erythrocytes [11,12], thus making interrogation of *P*. *knowlesi* cellular biology more accessible.

Malaria parasites export to the infected erythrocyte numerous self-encoded proteins which facilitate import of nutrients into the parasite and mediate erythrocyte surface exposition of ligands. Some members of the ‘exportome’ facilitate the establishment of parasite-induced structures in erythrocytes of varied morphology and physiology across *Plasmodium* [13]. In *P*. *falciparum*, the newly formed membranous structures termed Maurer’s clefts (MCs) are thought to be protein marshaling platforms where exported proteins, such as the Pfmc-2TM integral membrane proteins [14], either reside or transiently associate en route to the erythrocyte membrane [15]. The well characterized *P*. *falciparum* skeleton-binding protein 1 (PfSBP1) is an exported integral membrane protein that resides on the MC membrane. PfSBP1 has been shown to participate in protein interactions that anchor MCs to the erythrocyte cytoskeleton [16,17]; however, the absence of PfSBP1 does not affect the number, morphology, and position of MCs [18,19]. PfSBP1 is not involved in the trafficking mechanism of some MC-associated proteins, such as ring exported protein-1 (REX1) [20], membrane-associated histidine-rich protein 1 (MAHRP1) [21,22], and knob-associated histidine-rich protein (KAHRP) [23], but is required for transport of erythrocyte surface variant ligand termed *P*. *falciparum* erythrocyte membrane protein 1 (PfEMP1) to the erythrocyte surface [18,19]. Surface display of PfEMP1 is also affected by the absence of proteins like MAHRP1 [22], PfEMP3 [24] as well as the soluble KAHRP [23] implying that the role of PfSBP1 in the erythrocyte surface display of the antigens is vital but not sufficient. The first *Plasmodium* gene family demonstrated to undergo antigenic variation was found in *P*. *knowlesi*; specifically, the Schizont Infected Cell Agglutination variant antigen (*SICAvar*). This protein shares an intracellular tryptophan-rich motif, and might be analogous in function to the *P*. *falciparum* erythrocyte surface ligand PfEMP1 [25–27]. Recently, the SBP1 ortholog (PbSBP1) of the rodent malaria parasite *Plasmodium berghei* and some other *Plasmodium* species were reported [28]. PbSBP1 localizes to the dot-like structures in the cytoplasm of the infected erythrocytes. Disruption of PbSBP1 resulted in the reduced activity of the cytoadhesion, which was restored by the complementation of *P*. *falciparum* SBP1, indicating that the dot-like structures in *P*. *berghei*-infected erythrocytes are functionally equivalent to the Maurer's clefts in *P*. *berghei*-infected erythrocytes. However, no PfSBP1 orthologs have been characterized in *P*. *knowlesi* and it is not clear whether *P*. *knowlesi* possess functionally equivalent MC-like structures, given that the morphologies of parasite-induced erythrocyte structures vary across species [13,29].

To better understand the structures and mechanisms underlying *Plasmodium* protein export, we have further analyzed SBP1 orthologs in other *Plasmodium* species. Using the zoonotic *P*. *knowlesi*, we explore the hitherto unknown protein export structures and mechanism by examining the localization of the *P*. *knowlesi* SBP1 (PkSBP1) ortholog which is likely similarly important to PfSBP1 and PbSBP1, for which gene knockouts have been performed [18,28].

## Results

### Analysis of SBP1 orthologs

**Fig 1:**
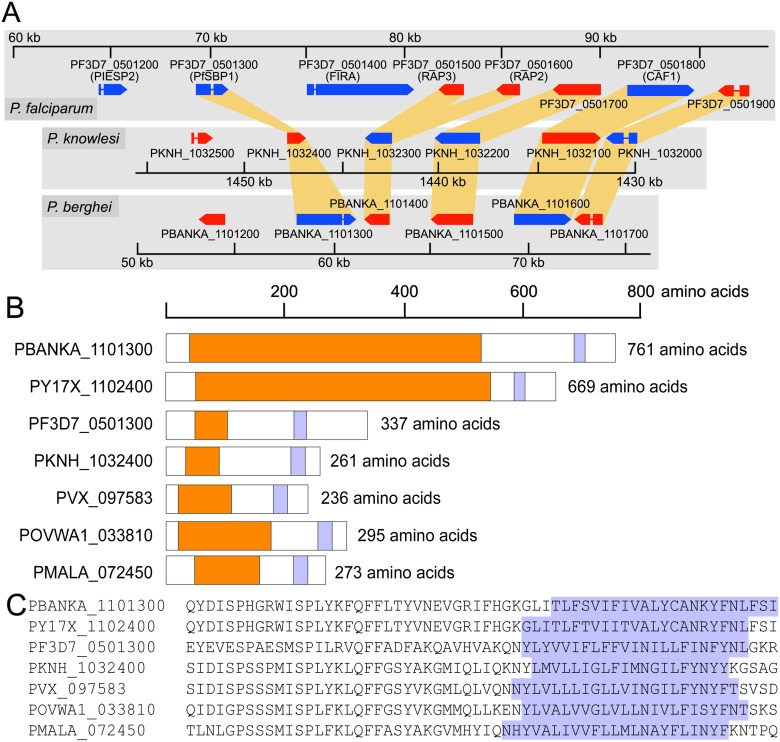
Conservation of *sbp1* gene synteny. **(A)** Genomic information corresponding to 30 to 50 kb of the left arm of *P*. *falciparum* chromosome 5 (top), the right arm of *P*. *knowlesi* chromosome 10 (middle) and the left arm of *P*. *berghei* chromosome 11 (bottom). Syntenic genes are highlighted in orange. To better represent synteny, the *P*. *knowlesi* genome neighborhood is inverted, but the original color coding of genes encoded on the top strand (blue) and bottom strand (red) and genome position (represented by number scales for each species) have been retained. **(B)** Schematics of *Plasmodium* SBP1 orthologs showing the overall length and general protein structures. Repeat regions are shown in orange and the single transmembrane regions in light blue. Recently reported SBP1 orthologs of *P*. *ovale* and *P*. *malariae* are also included. **(C)** The conservation of amino acid sequence within the transmembrane and adjacent regions. The transmembrane regions are predicted by TMHMM 2.0 and highlighted in light blue.

A candidate SBP1 protein for *P*. *knowlesi* was identified by BLASTP analysis using the *P*. *falciparum* SBP1 amino acid sequence as a query of GenBank and the *Plasmodium* genome databases accessed at PlasmoDB independently from the recent report by de Niz *et al*. (2016) [28]. Candidate SBP1 proteins for other *Plasmodium* were identified by the same manner using the putative *P*. *knowlesi* SBP1 sequence. Because of the low similarity in amino acid sequences and lack of signature domains or motifs, orthology was confirmed by the observation of conservation of synteny for the predicted *sbp1* genes within their extended loci across *Plasmodium* (Fig 1A). To this end, *sbp1* was identified to be conserved as a single copy gene across the *Plasmodium* genus. SBP1 proteins share a general protein structure highlighted by the absence of a signal peptide sequence and *Plasmodium* Export Element (PEXEL) or host targeting (HT) erythrocyte targeting motif [30,31], the presence of a central low complexity or repeat region (Fig 1B), and amino acid sequence similarity confined to a region adjacent to the single transmembrane region (Fig 1C). The central region varies in length and composition of repeat motifs. For example, in *P*. *berghei* SBP1 the central region spans roughly 500 amino acids (aa) and is composed of a tandem array of a 14 aa long repeat; whereas the *P*. *knowlesi* version lacks recognizable repeats and instead has a proline and small, polar aa rich character of approximately 100 aa length (Fig 1B). In *P*. *falciparum* the repeat region is approximately 80 aa long and has a glycine-rich character. Similar results were obtained recently [28].

### Recombinant PkSBP1 (rPkSBP1) is exported to discrete structures in *P*. *knowlesi*-infected monkey erythrocytes

**Fig 2:**
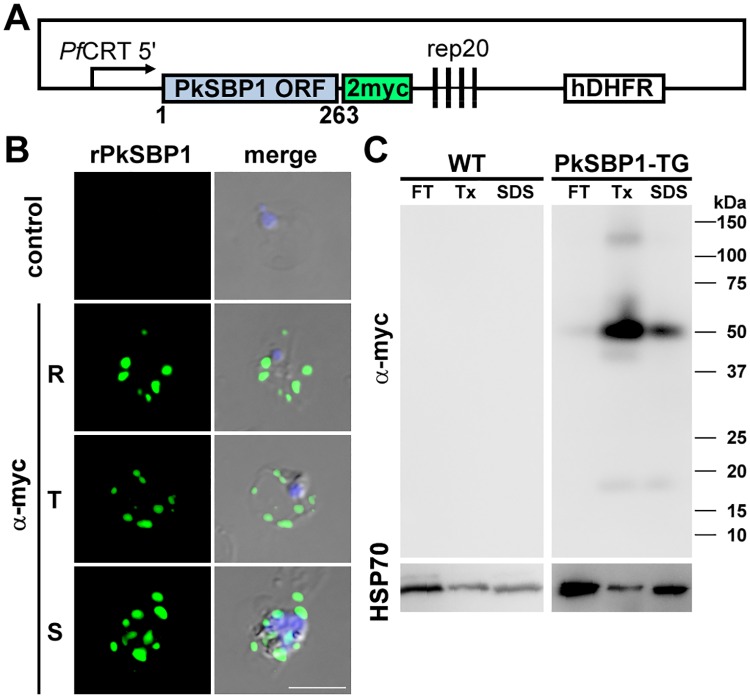
Expression and localization of rPkSBP1 in infected monkey erythrocytes. **(A)** Schematic of *P*. *knowlesi* rPkSBP1 expression construct (not to scale). Two myc epitopes (2myc) were fused at the C-terminus of full-length PkSBP1 open reading frame (PkSBP1 ORF) and expressed using the *P*. *falciparum* CRT 5' region (PfCRT 5') as a promoter. **(B)** Representative IFAT images of PkSBP1-transgenic *P*. *knowlesi* H-DMU line with anti-myc antibody (α-myc, green). α-myc-stained rPkSBP1 images were merged with DAPI nucleus-staining (blue) and differential interference contrast image (merge). The top panel is a negative control reacted with normal mouse IgG. R, ring; T, trophozoite; S, schizont stages. Scale bar represents 5 μm. **(C)** Western blotting of wild type parental *P*. *knowlesi* H-DMU line (WT) and PkSBP1-transgenic line (TG) with anti-myc antibody. Parasite proteins were sequentially extracted by freeze-thawing (FT), followed by extraction with 1% Triton X-100 (Tx), then with 2% SDS. Parasite protein cross-reacting by an antibody against *P*. *berghei* HSP70 serves as a loading control (bottom).

To describe the modification of host erythrocytes by *P*. *knowlesi* we developed PkSBP1 as cellular markers by constructing plasmids expressing chimeras of sequences encoding full-length PkSBP1 fused to 2 myc epitopes at the C-terminus, to give rPkSBP1. The PkSBP1 nucleotide sequence obtained from the H-DMU line (accesion number LC155843) was different from the sequence in the database (strain H; AM910992) with one codon deletion and 18 substitutions (S1 Fig). The fragment was inserted into the transfection vector pCHD43(II) under the control of the PfCRT 5’ region as a promoter (Fig 2A). Transgenic parasites were obtained by episomal transfection to *P*. *knowlesi* (H-DMU line)-infected monkey erythrocytes. We then used imaging tools to demonstrate that chimeric protein is expressed and transported to the erythrocyte cytoplasm following infection of monkey erythrocytes. To evaluate if rPkSBP1 is expressed, asynchronous parasite cultures of parent *P*. *knowlesi* and transgenic lines were subjected to IFAT by staining with mouse anti-myc antibody. Fluorescence microscopy of the parent *P*. *knowlesi* line showed no reactivity, as was the case for uninfected erythrocytes. The rPkSBP1 was observed at all parasite stages as a punctate staining pattern in the cytoplasm of infected monkey erythrocytes (Fig 2B). The stained puncta containing rPkSBP1 occasionally appeared to be peripheral and closely associated with the host cell membrane, especially at the trophozoite and schizont stages. The localization indicates that rPkSBP1 is expressed and exported to the infected monkey erythrocyte cytoplasm.

The primary structure of PkSBP1 protein contains a predicted transmembrane region. To evaluate whether PkSBP1 is an integral membrane protein, we performed differential extractions of infected erythrocytes and separation of the protein extracts via SDS-PAGE followed by Western immunoblotting with anti-myc antibody. A major band of approximately 50 kDa was detected in the Tx-soluble and Tx-insoluble fractions of rPkSBP1-expressing parasites, which was greater in molecular weight than the calculated full-length chimera (31.5 kDa; Fig 2C). Also observed in the Tx fraction were an approximately 130-kDa band, a potential multimeric protein complex containing rPkSBP1; and other smaller bands at roughly 40 and 20 kDa, most likely representing processed products. PkSBP1 likely associates in membranes during blood stage infection because the majority of the rPkSBP1 was not released in the water soluble fraction, but rather in the Tx fraction. The rPkSBP1 band present in the water soluble fraction might be a soluble trafficking intermediate.

### rPkSBP1 is exported to Maurer's clefts in *P*. *falciparum* and ‘Sinton and Mulligan’ stipplings in *P*. *knowlesi*

**Fig 3:**
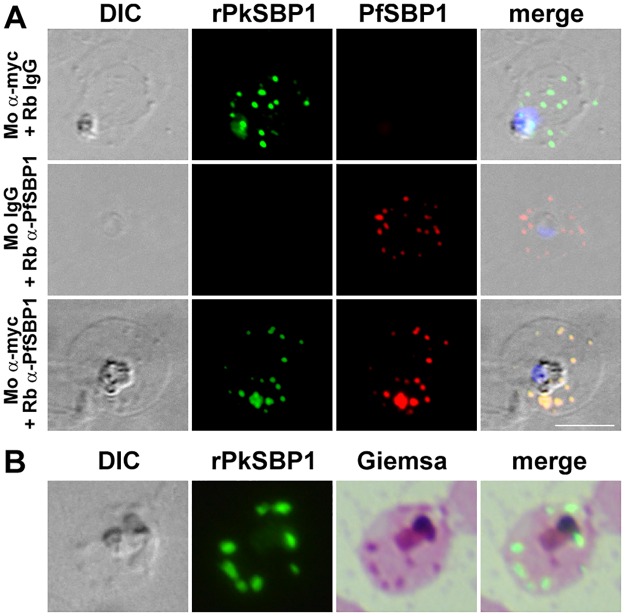
rPkSBP1 is exported to *P*. *falciparum* Maurer's clefts and *P*. *knowlesi* ‘Sinton and Mulligan’ stipplings. **(A)** Human erythrocytes infected with PkSBP1-transgenic *P*. *falciparum* co-stained with anti-myc antibody (green) and PfSBP1 (red). Merged image of rPkSBP1, PfSBP1, DAPI nucleus-staining (blue), and differential interference contrast (DIC) image are shown (merge). Top panel was labeled with anti-myc antibody (α-myc) and normal rabbit IgG, middle panel was labeled with normal mouse IgG and rabbit anti-PfSBP1 antibody, and bottom panel was labeled with mouse anti-myc and rabbit anti-PfSBP1 antibodies. **(B)** Colocalization of rPkSBP1 puncta (green) and Giemsa-stained ‘Sinton and Mulligan’ stipplings in monkey erythrocytes infected with PkSBP1-transgenic *P*. *knowlesi* H-DMU line. Merged image of rPkSBP1 and Giemsa-stained image are shown (merge). Scale bar represents 5 μm. Nuclei were stained with DAPI (blue).

The relationship between the rPkSBP1-stained puncta and Maurer’s clefts in the infected human erythrocyte was examined by co-labelling transgenic *P*. *falciparum* parasites with anti-PfSBP1 and anti-myc antibodies. The antibody recognizing endogenous PfSBP1 showed complete overlap of rPkSBP1-positive puncta and PfSBP1-stained Maurer’s clefts in the cytoplasm of the infected erythrocyte (Fig 3A). Thus, rPkSBP1 in *P*. *falciparum* is trafficked to the Maurer’s clefts similarly to endogenous SBP1. Because no cellular markers have been described for *P*. *knowlesi*-infected erythrocytes, we used IFAT with anti-myc antibody and Giemsa dual-labelling to examine the identity of stained structures in *P*. *knowlesi*-infected monkey erythrocytes (Fig 3B). The rPkSBP1-positive puncta co-localized with Giemsa-stained structures, described as ‘Sinton and Mulligan’ stipplings [32], at trophozoite stage. This data suggests that the ‘Sinton and Mulligan’ stipplings of monkey erythrocytes infected with *P*. *knowlesi* are likely functionally analogous to Maurer’s clefts.

### Ultrastructural analysis of *P*. *knowlesi*-infected monkey erythrocyte

**Fig 4:**
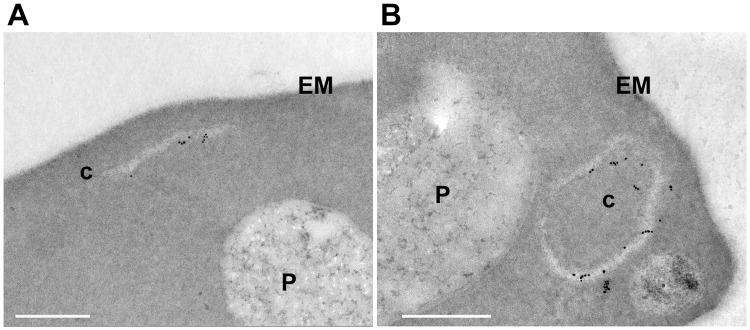
rPkSBP1-positive membranous structures in the monkey erythrocytes infected with PkSBP1-transgenic H-DMU line. Representative micrographs of immunogold-labeled rPkSBP1. Gold particles were visible at slit-like clefts (**A**) and oblong vesicular clefts (**B**) in the erythrocyte cytoplasm infected with PkSBP1-transgenic line. c, clefts; EM, erythrocyte membrane; P, parasite. Scale bar represents 500 nm.

To reveal the fine structure of modifications induced by *P*. *knowlesi* infection to monkey erythrocytes, we used immunoelectron microscopy to examine the subcellular location of rPkSBP1. We observed membranous structures that were decorated with gold particles of two profiles; namely, slit-like unilamellar and oblong vesicular cleft structures (Fig 4). We did not detect these membranous structures in uninfected erythrocytes and gold particles were not detected in the monkey erythrocytes infected with wild type *P*. *knowlesi* (S2 Fig). This data taken together with IFAT-Giemsa dual-labelling suggests that the gold particle-positive membranous structures are identical to the ‘Sinton and Mulligan’ stipplings.

### Full-length rPk2TM-a is expressed and exported to discrete structures in *P*. *knowlesi*-infected monkey erythrocytes

**Fig 5:**
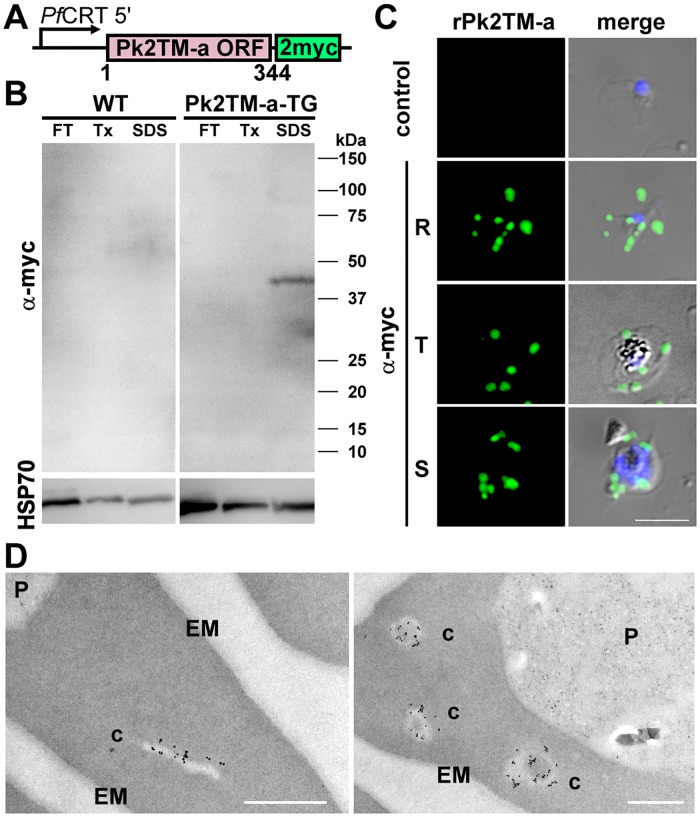
Expression and localization of rPk2TM-a in the monkey erythrocytes infected with Pk2TM-a-transgenic *P*. *knowlesi* H-DMU line. **(A)** Schematic of the expression cassette of the *P*. *knowlesi* rPk2TM-a (not to scale). Two myc epitopes (2myc) were fused at the C-terminus of full-length Pk2TM-a open reading frame (Pk2TM-a ORF) and expressed using the *P*. *falciparum* CRT 5' region (PfCRT 5') as a promoter. Plasmid backbone is shown in Fig 2A. (**B**) Western blotting of wild type parental *P*. *knowlesi* H-DMU line (WT) and Pk2TM-a-transgenic *P*. *knowlesi* H-DMU line (TG) with anti-myc antibody (α-myc). Parasite proteins were sequentially extracted by freeze-thawing (FT), followed by extraction with 1% Triton X-100 (Tx), then with 2% SDS. Parasite protein cross-reacting by an antibody against *P*. *berghei* HSP70 serves as a loading control (bottom). **(C)** Representative IFAT images of Pk2TM-a-transgenic *P*. *knowlesi* parasites with anti-myc antibody (α-myc, green). α-myc-stained rPk2TM-a images were merged with DAPI nucleus-staining (blue) and differential interference contrast image (merge). The top panel is a negative control. R, ring; T, trophozoite; S, schizont stages. Scale bar represents 5 μm. **(D)** Representative transmission electron micrographs of immunogold labeled Pk2TM. Slit-like clefts (left) and oblong vesicular clefts (right) showed gold particles in the erythrocyte cytoplasm infected with Pk2TM-a-transgenic line. c, clefts; EM, erythrocyte membrane; P, parasite. Scale bar represents 500 nm.

Localization of rPkSBP1 at MCs in *P*. *falciparum* prompted us to examine the location of another putative MC-resident protein, a member of the Pk2TM family, whose possible homolog in *P*. *falciparum*, Pfmc-2TM, is known to be exported to the surface of infected erythrocyte via MCs [33,34]. To this end, we generated a transgenic *P*. *knowlesi* H-DMU line expressing recombinant Pk2TM-a (rPk2TM-a) fused to 2 myc epitopes using a plasmid constructed similarly as the one for rPkSBP1. The Pk2TM-a nucleotide sequence obtained from the H-DMU line (accession number LC155844) was different from the sequence in the database (strain H; PKNH_0623600) with 4 substitutions (S1B Fig). To confirm expression of rPk2TM-a, we performed Western immunoblotting with anti-myc antibody for parasite extracts, as described above for rPkSBP1. A single band in the Tx-insoluble fraction was detected at about 41 kDa in the rPk2TM-a-expressing parasites (Fig 5A), suggesting that rPk2TM-a tightly associated with the membrane. IFAT images showed a punctate staining pattern in the cytoplasm of the erythrocyte infected with the Pk2TM-a-transgenic line (Fig 5B). IEM detected rPk2TM-a on the membranous structures in the erythrocyte cytoplasm, similar to those detected for rPkSBP1 (Fig 5C). However, anti-myc antibody did not detect signals at the surface membrane of infected erythrocyte.

### *P*. *knowlesi* induces similar modifications in monkey and human erythrocytes

**Fig 6:**
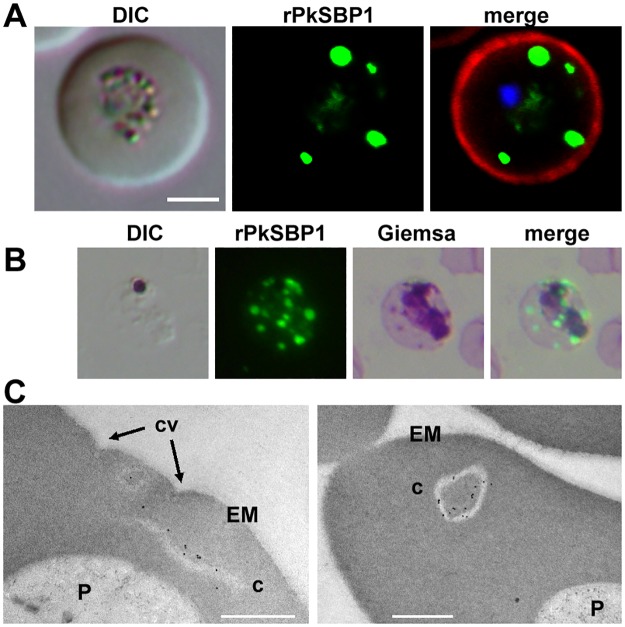
Expression and localization of rPkSBP1 in the human erythrocytes infected with PkSBP1-transgenic *P*. *knowlesi* H_hu_-HSPH line. **(A)** Representative fluorescence image showing localization of rPkSBP1 stained with anti-myc antibody (rPkSBP1, green) as puncta within the infected erythrocyte cytoplasm. rPkSBP1 signal was merged with erythrocyte membrane stained with anti-human CD235a (α-GlyA, red) and DAPI nucleus-staining (blue) (merge). DIC, differential interference contrast. Scale bar represents 5 μm. **(B)** Colocalization of rPkSBP1 puncta (green) and Giemsa stained ‘Sinton and Mulligan’ stipplings in the human erythrocyte infected with PkSBP1-transgenic *P*. *knowlesi* H_hu_-HSPH line. Merged image of rPkSBP1 and Giemsa-stained image are shown (merge). Scale bar represents 5 μm. **(C)** Transmission electron micrographs of two representative erythrocytes infected with PkSBP1-transgenic *P*. *knowlesi* H_hu_-HSPH line. Slit-like clefts (left) and oblong vesicular clefts (right) were observed. c, clefts; cv, caveola; EM, erythrocyte membrane; P, parasite. Scale bar represents 500 nm.

To examine *P*. *knowlesi* modification of the human erythrocyte, we expressed rPkSBP1-2myc in the *P*. *knowlesi* H_hu_-HSPH line that was adapted to human erythrocytes. IFAT images of the transgenic parasites showed that the chimeric protein distributed as puncta in the cytoplasm of the infected human erythrocyte similar to the ones observed in the parasite-infected monkey erythrocyte (Fig 6A). To discern the organization of the puncta and the erythrocyte membrane, rPkSBP1 and erythrocyte membrane were stained with anti-myc antibody and rat monoclonal anti-CD235a antibody, respectively; and confocal microscopy imaging was performed to obtain consecutive optical slices of the transgenic parasites expressing the rPkSBP1. 3D reconstructions of the slices (S1 Video) revealed puncta containing rPkSBP1 chimera close to the periphery of infected human erythrocytes. These structures might be tethered to the cytoskeleton as shown for *P*. *falciparum* MCs [17]. There were ‘Sinton and Mulligan’ stipplings in infected human erythrocytes as shown by IFAT-Giemsa dual-labelling (Fig 6B). We did not observe any difference in the number of ‘Sinton and Mulligan’ stipplings in infected human erythrocytes compared to that in infected monkey erythrocytes (S3 Fig). The subcellular location of rPkSBP1 by IEM revealed a similar staining pattern of two membrane profiles as observed in infected monkey erythrocytes (Fig 6C). Antibody labeling was not observed in the wild type parental *P*. *knowlesi* H_hu_-HSPH line-infected human erythrocyte (S4 Fig) and surface structures termed caveolae in PkSBP1-transgenic *P*. *knowlesi* H_hu_-HSPH line (Fig 6C). These data indicate that the morphology and number of the rPkSBP1-positive membranous structures generated in two distinguished host erythrocytes are similar and determined by the parasite, and are not influenced by possible differences in host cell factors.

## Discussion

The recognition of human knowlesi malaria has allowed the opportunity to understand the mechanisms underlying the host switch from primate to human hosts [35,36]. Successful infection and adaptation are determined by the biology of both the host and parasite. This study sought to examine how *P*. *knowlesi* modifies host erythrocytes and to determine whether adaptation to humans presented differential or new host cell modifications. Overall, we provide evidence that *P*. *knowlesi* erythrocyte modifications are determined by the parasite and not influenced by the host cell factors. In our study based on comparative bioinformatics, we have analyzed *sbp1* orthologs in all *Plasmodium* species for which genome sequence information is available, including recently reported *Plasmodium ovale* and *Plasmodium malariae* [37]. *P*. *knowlesi* SBP1 is exported to the erythrocyte and localizes to the ‘Sinton and Mulligan’ stipplings of the infected erythrocyte. In *P*. *falciparum*, the mechanism of PfSBP1 transport across the parasitophorous vacuole membrane into the erythrocyte cytoplasm is via a translocon although PfSBP1 lacks a signal peptide and PEXEL/HT motif [38,39]. Transport of PfSBP1 beyond the parasitophorous vacuole membrane to the Maurer’s clefts is unclear and is possibly via vesicular trafficking or transport as a protein complex. Our findings provide evidence that PkSBP1 is exported in a similar manner to PfSBP1, and the transmembrane nature of PkSBP1 implicates rPkSBP1-positive puncta as membranous structures. The C-terminal domain of PfSBP1 is exposed to the erythrocyte cytoplasm, and it participates in protein interactions at the erythrocyte cytoskeleton [16,17]. We did not observe any C-terminal domain homology among SBP1 orthologs, including PfSBP1 and PkSBP1; thus the functional motifs of PkSBP1 remain to be investigated.

Previous ultrastructure studies of *P*. *knowlesi*-infected monkey erythrocytes observed narrow slit-like membranous structures [13,40]. Our analyses revealed more detail; specifically, in addition to the already described clefts, we detected oblong vesicular cleft structures in both infected monkey and human erythrocytes. We found both structures decorated with gold particle-labeled antibody, indicating that PkSBP1 and Pk2TM-a are resident on these membranes. Based on our observations, the protein composition between the slit-like and oblong vesicular membranous structures was similar, suggesting that a similar mechanism incorporates the two proteins into the structures, and/or the structures are formed by a similar process. It is formally possible that the slit-like structures and oblong vesicles observed in the *P*. *knowlesi*-infected erythrocyte correspond to the same type of structure that was just sectioned at different angles. However, oblong vesicular clefts are documented and are distinct from the slit-like membranous structures in *P*. *falciparum*-infected erythrocytes [41, 42] and oblong vesicles in *P*. *knowlesi*-infected erythrocytes appear consistently bigger than slit-like structures in majority of the ultrathin-sections collected, thus we speculate that these two types of membranous structures are distinct also for *P*. *knowlesi*. It should be noted that although the morphology is similar between these two species, to our knowledge PfSBP1 localization on the oblong vesiclar structures has not yet been reported.

Using *P*. *falciparum* as a surrogate system, successful heterologous expression and correct export of rPkSBP1 to *P*. *falciparum* MCs suggests that rPkSBP1 puncta mark structures homologous to MCs. Our data correlated the ‘Sinton and Mulligan’ stipplings with rPkSBP1-positive puncta when infected erythrocytes were co-labelled with Giemsa and immunofluorescent antibodies. Colocalization of the stipplings with rPkSBP1 puncta shows that the stipplings contain or are composed of proteins. Thus, we speculate the role of ‘Sinton and Mulligan’ stipplings to be identical to the MCs and propose to term the membranous structures positive for rPkSBP1 as Sinton and Mulligan's clefts. Taken together with findings from PfSBP1 studies, our results of rPkSBP1 and rPk2TM-a propose a model that parasite-encoded proteins are trafficked to the erythrocyte membrane via membranous structures generated by the parasite in the erythrocyte cytoplasm and this system is conserved between *P*. *falciparum* and *P*. *knowlesi*. In accordance with this, a recent report indicated that *P*. *falciparum* SBP1 was able to complement the function of its ortholog in *P*. *berghei* to modify the erythrocyte cytoadherence property [28].

Ultrastructural analyses revealed some difference in the organization of *P*. *knowlesi*-induced membranous structures from the MCs. In *P*. *falciparum*, MCs connect to the erythrocyte membrane through tether structures [21]. Occasionally, we observed rPkSBP1-positive membranous structures beneath the infected erythrocytes membrane, but most of these structures localized distant from the infected erythrocytes surface. Accessory tether structures may then be involved in connecting the rPkSBP1-positive membranes to the erythrocyte cytoskeleton to maintain a spatial relationship enabling PkSBP1 interaction with erythrocyte cytoskeleton. This hypothesis remains to be tested in *P*. *knowlesi*.

We did not observe differences in erythrocyte modification upon *P*. *knowlesi* infection to the monkey and human erythrocytes; partly as observed by enumeration of rPkSBP1-positive puncta. Moreover, the shape, size, and distance of membranous structures from the infected erythrocytes membrane observed by TEM were comparable between infected monkey and human erythrocytes. Albeit, caution should be exercised when interpreting ultrathin sections from infected erythrocytes as they represent only two-dimensional detail from a single section. More detailed 3D analysis of the host cell modification by *P*. *knowlesi* needs to be further investigated. Nonetheless, these findings suggest that overall emerging zoonotic simian *Plasmodium* species are not presenting distinct protein transport pathways between natural host and human erythrocytes. While different studies have established the importance of PfSBP1 in the exposure of the virulence-related PfEMP1 on the infected erythrocytes surface and PbSBP1 in the cytoadherence activity [28], the role of the PkSBP1 remains to be explored.

**Fig 7:**
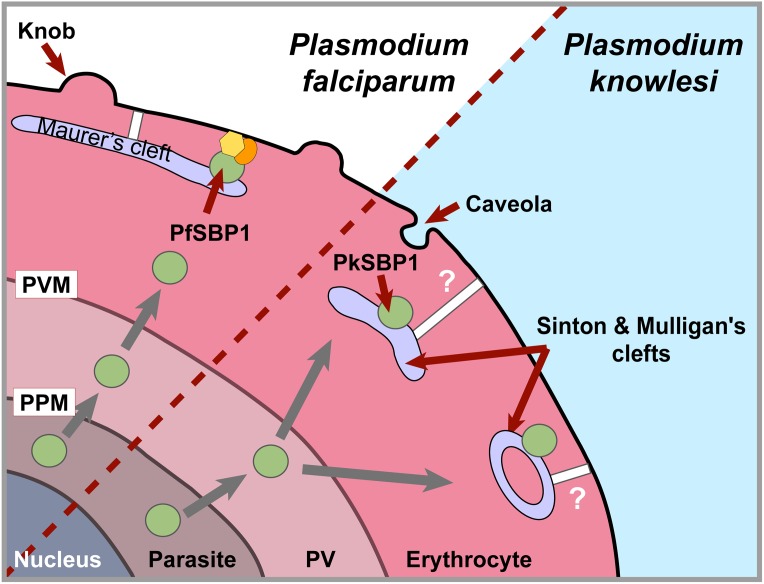
PkSBP1 delineates *P*. *knowlesi* host modifications. Schematic representation of host erythrocyte modifications revealed by studying the localization of SBP1 ortholog. *P*. *knowlesi* infection in both monkey and human erythrocytes induces membranous structures onto which PkSBP1 localizes. Involvement of tether structures (white bars) adjoining these membranes to the host cell cytoskeleton is possible [21] but was not investigated in this study. PfSBP1 interacts with erythrocyte membrane protein 4.1 and spectrin, as described for *P*. *falciparum* (yellow and orange shapes) [16]. PPM, parasite plasma membrane; PV, parasitophorous vacuole; PVM, parasitophorous vacuole membrane.

In summary, we have established markers for membranous structures in *P*. *knowlesi* by examining the localization of a newly identified SBP1 ortholog, as well as a Pk2TM protein. We have provided evidence for the similarity of the export apparatus in terms of the existence of SBP1-positive membranous structures, with a difference in the relative location from the erythrocyte membrane between *P*. *falciparum* and *P*. *knowlesi* (as schematized in Fig 7). Our data further strengthens the hypothesis that protein export pathways are evolutionarily conserved in the genus *Plasmodium*. *Plasmodium* protein export structures and mechanisms can be intervention targets since malaria parasites are obligate intracellular parasites that must establish new secretory pathways to transport materials across membranes and the host’s cytoplasm. Intervention strategies aimed at disruption of protein export structures in the host cell augur inhibition of exposition of virulence ligands at the surface of infected erythrocytes in *Plasmodium*.

## Materials and Methods

### *In silico* identification of PfSBP1 orthologs and Pk2TM-a

The PfSBP1 (PF3D7_0501300) amino acid sequence without repeats was used as a query in the Basic Local Alignment Search Tool (BLAST) [43] against GenBank and PlasmoDB (http://plasmodb.org) protein databases; hence identifying a possible ortholog in the *P*. *knowlesi* genome. Further BLASTP search using the putative PkSBP1 ortholog sequence identified putative orthologs in the *P*. *berghei*, *P*. *vivax*, *P*. *yoelii*, *P*. *ovale* and *P*. *malariae* genomes [37]. Annotation of the genes within the extended loci surrounding retrieved *sbp1* genes allowed confirmation of a conserved gene synteny across *Plasmodium* and thereby supporting the prediction of SBP1 orthology. The protein PKNH_0623600 (former PKH_062310; termed Pk2TM-a in this study) of the 2TM family was identified from annotation of the subtelomeric regions of the *P*. *knowlesi* genome [44]. Signal peptides were identified using SignalP [45] and transmembrane regions were predicted using TMHMM 2.0 [46]. PEXEL/HT erythrocyte targeting motifs were identified by eye.

### Malaria parasites

The *P*. *knowlesi* H strain was obtained from ATCC (ATCC No. 30158) and maintained in Japanese macaques (*Macaca fuscata*) in Dokkyo Medical University, Japan, herein termed the "H-DMU" line, and was adapted to propagate *in vitro*. These monkeys were second-generation offspring bred in captivity. All monkeys were bred and grown at animal facilities in a malaria-free environment in Japan, housed in individual cages (cage size: 650[W] x 750[D] x 820[H] mm) in a controlled environment (22–25°C and 30–60% humidity and 12- hour light/dark cycle). They were fed a standard diet for the old world monkey supplemented with fresh fruits and given water *ad libitum*. The investigators adhered to the Guidelines for the Use of Experimental Animals authorized by the Japanese Association for Laboratory Animal Science. The protocol was approved by the Committee on the Ethics of Animal Experiments of the Dokkyo Medical University (Permit Number: 0656). The monkeys were intravenously inoculated with frozen stock of *P*. *knowlesi*-infected erythrocytes. For hematological examination, venous blood samples were obtained every 3 or 4 days after inoculation. These procedures were performed under anesthesia with a combination of ketamine hydrochloride (10 mg/kg, i.m.) and xylaxine (0.5 mg/kg, i.m.), and all efforts were made to minimize suffering. From day 1 after inoculation, Giemsa-stained thin blood films were prepared daily from peripheral blood obtained by earprick, and development of parasites in the infected monkeys was monitored by microscopic observation. After parasite inoculation, monkeys were monitored daily by a veterinarian during the experiment for clinical signs (e.g., changes in respiration and appetite), body condition (visual inspection of the obesity and leanness), and well-being (e.g., changes of fur and a lack of atypical behavior). The humane endpoint for the infectious experiment was determined when parasitemia in the peripheral blood reached to 5%, and all animals were exsanguinated under deep anesthesia by the combination of ketamine hydrochloride/xylaxine/pentobarbitone (30 mg/kg, i.v.). The details of animal welfare/care and steps taken to ameliorate suffering were in accordance with the recommendations of the Weatherall report “The use of non-human primates in research”. Human erythrocyte-adapted *P*. *knowlesi* H strain, herein termed the "H_hu_-HSPH" line, was provided by M. Duraisingh (Harvard T. H. Chan School of Public Health, MA, USA) [11]. The *P*. *falciparum* MS822 field isolate from Mae Sot, Thailand was described before [47].

### Culture conditions for malaria parasites

*P*. *knowlesi* H-DMU line parasites were cultivated in RPMI-1640 medium supplemented with 0.5% AlbuMAX lI (Invitrogen, Carlsbad, CA), 200 mM hypoxanthine (Sigma, St. Louis, MO), 20 μg/ml gentamicin (Invitrogen) and rhesus macaque erythrocytes at 2% hematocrit. Usage of rhesus monkey blood was approved by the Animal Care and Use Committee, the Corporation for Production and Research of Laboratory Primates, Japan, and the monkey blood were provided from Non-human Primate Reagent and Resource Program in Tsukuba Primate Research Center, National Institute of Biomedical Innovation, Japan. *P*. *knowlesi* H_hu_-HSPH line parasites were cultivated under similar conditions as above except using O^+^ human erythrocytes. *P*. *falciparum* was maintained in RPMI-1640 medium containing 5% heat-inactivated pooled type AB^+^ human serum, 0.25% AlbuMAX I (Invitrogen), 200 mM hypoxanthine, 20 μg/ml gentamicin and O^+^ human erythrocytes at 2% hematocrit.

### Plasmid construction

The PKNH_1032400 gene (former ID PKH_103200) was identified as encoding PkSBP1 as above and was amplified from *P*. *knowlesi* H-DMU line genomic DNA using KOD -Plus- DNA polymerase (Toyobo, Japan) with primers PKH_103200_s (GG**GCTAGC**TAAAAAATGTGTCAAGCATTTGCAAGT) and PKH_103200_as (GT**GGTACC**AATGGCATCTCCATCGAGTT). The PKNH_0623600 (Pk2TM-a) gene was amplified with PKH_062310_s (GG**GCTAGC**TAAAAAATGAAAGAAATATTTCTGTTC) and PKH_062310_as (GT**GGTACC**TTCTTTAATTTTAACACCGT). The primer pairs for each gene contained Nhel and KpnI sites (shown in bold). The purified PCR products were ligated into the Nhel and KpnI sites in pB1/3_2myc vector using T4 DNA ligase (Promega, Madison, WI). pB1/3_2myc is a pUC19-based plasmid containing attB1 and attB3. Using the Multisite Gateway system (Invitrogen) to prepare plasmids, BP recombination reactions of pDONR^™^P1-P3 (Invitrogen), which was generated from pDONR^™^221 (Invitrogen) by replacing attP2 motif to attP3 motif, with pB1/3_PkSBP1-2myc and pB1/3_Pk2TM-a-2myc were performed to obtain the entry clones pENTR1/3_PkSBP1-2myc and pENTR1/3_Pk2TM-a-2myc, respectively. The pENTR1/3-based plasmids were subjected to a Gateway Multisite LR recombination reaction with pENTR4/1_PfCRT5’ and pCHD43(II) [48,49]. The plasmids pENTR4/1_PfCRT5’ and pCHDR-3/4 (origin of pCHD43(II)) are gifts from G. McFadden (University of Melbourne, Australia). The plasmids obtained were verified by analyzing the restriction enzyme digestion pattern and nucleotide sequencing.

### Generation of transgenic parasites

Mixed asexual stages of *P*. *knowlesi* parasites were transfected using Amaxa Nucleofector 2b (Lonza, Switzerland) and Amaxa Human T cell kit (Lonza). Approximately 50 μg of plasmid DNA in incomplete cytomix (120 mM KCl, 0.15 mM CaCl_2_, 2 mM EGTA, 5 mM MgCl_2_, 10 mM K_2_HPO_4_, and 25 mM HEPES) was added to 100 μl of human T cell nucleofector solution. ~1x10^7^-10^8^ parasites were resuspended in the DNA-human T cell nucleofector solution mixture in Nucleofector kit cuvettes (Lonza) and electroporated using the U-33 program. The cuvettes were immediately put on ice and then the electroporated cells were transferred to a tissue culture flask containing 10 ml of 37°C-prewarmed complete medium and rhesus monkey or human erythrocytes to a final hematocrit of 2%. Three nanomolar of antifolate drug WR99210 (a gift from D. Jacobus, Jacobus Pharmaceutical Co. Inc., USA) was supplied in culture medium starting 24 hours post transfection. The drug pressure was maintained continuously. *P*. *falciparum* MS822 parasites were transfected using the erythrocyte loading method as described [50]. Briefly, human erythrocytes were suspended in 400 μl of incomplete cytomix containing 50 μg of plasmid DNA. Electroporation was performed in 2 mm cuvettes using the Gene Pulser Xcell Electroporation system (Bio-Rad, Hercules, CA) with a set program of 0.32 kV and 950 μF. Mixed stage parasite culture was resuspended with plasmid-preloaded erythrocytes (final parasitemia 0.1%). Five nanomolar WR99210 was supplied in culture medium starting on day 4 post transfection until drug-resistant parasites appeared, then parasites were maintained with 10 nM WR99210 henceforth.

### Immunofluorescence microscopy

For indirect immunofluorescence antibody test (IFAT) thin blood films on glass slides were prepared from mixed stage cultures with 3–10% parasitemia, air-dried at room temperature (RT), and kept at −80°C till further use. Retrieved blood films were thawed at RT in a silica gel desiccator and were fixed at RT for 15 min using 4% paraformaldehyde (Nacalai Tesque, Japan) and 0.075% glutaldehyde (Nacalai Tesque, Japan) in phosphate-buffered saline (PBS). The reaction was neutralized with 50 mM glycine (Wako, Japan) in PBS for 10 min and immediately blocked with PBS containing 10% normal goat serum (Invitrogen) at 37°C for 60 min. Blood films were immunostained with mouse anti-myc monoclonal primary antibody (final 1:1000; 9B11, Cell Signaling Technology, Danvers, MA) and incubated at 37°C for 60 min. After washing thrice with PBS, the blood films were incubated with a solution containing Alexa fluor^®^ 488-conjugated secondary goat anti-mouse IgG antibody (final 1:500; Invitrogen) at 37°C for 30 min. Double immunostaining of the blood films with either mouse anti-myc antibody and rabbit anti-PfSBP1 antibody (provided by T. Tsuboi, Ehime University, Japan), or mouse anti-myc antibody and rat anti-human CD235a (glycophorin A; final 1:1000; YTH 89.1, Serotec, UK) were performed at 37°C for 60 min; the excess antibody was washed off with PBS. The blood films were incubated with solutions containing a selection of secondary Alexa fluor^®^ 488-conjugated goat anti-mouse, Alexa fluor^®^ 594-conjugated goat anti-rabbit or Alexa fluor^®^ 594-conjugated goat anti-rat (final 1:500; Invitrogen) IgG antibodies at 37°C for 60 min. Normal mouse and normal rabbit IgG (Merck Millipore, Germany) were used as negative controls. Nuclei were stained by incubating blood films with 4’,6-diamidino-2-phenylindole (DAPI, final 0.5 μg/mL; Invitrogen). ProLong^®^ Gold antifade reagent (Invitrogen) was used as a mounting solution. Alternatively, instead of thin blood films, cells fixed within a tube were permeabilized with 0.1% Triton X 100 in PBS and reacted with antibodies. Cells were let to settle on glass slides and gently covered with a mounting solution and coverslip.

Visualization of images was performed using either a fluorescent microscope (Axio Imager Z2; Carl Zeiss, Germany) equipped with a 100x/1.4 oil immersion lens and a CCD camera (AxioCam MRm; Carl Zeiss), or a laser scanning confocal microscope system (LSM 780 ELYRA system, Carl Zeiss). Some acquired images were processed and analyzed using Adobe Photoshop CS (Adobe Systems Inc. San José, CA) or IMARIS processing software (Version 7.2 Bitplane Inc, Switzerland) for 3D reconstruction. To enumerate the number of anti-myc antibody-positive dots, IFAT images were counted for 30 randomly selected transgenic parasites expressing recombinant PkSBP1-2myc (rPkSBP1) in the monkey or human erythrocytes. The median value for each parasite was determined, and statistical differences were evaluated by Mann—Whitney *U* test implemented in GraphPad PRISM^™^ 6 (GraphPad Software, Inc., San Diego, CA).

For IFAT-Giemsa co-staining, monolayer blood films were prepared for IFAT and images were acquired as in the IFAT section above. During image acquisition, the mechanical stage was used to record vernier coordinates (precision ± 0.1 mm) of parasites whose fluorescent images had been acquired. Coverslips were removed from the glass slides, the mounting solution was removed by washing the slides with methanol for 30 seconds followed by 2 min wash with PBS. The slides were then stained with 10% Giemsa azur eosin methylene blue solution (Merck Millipore) in phosphate buffer (pH 6.8) for 90 min. The slides were washed with water for 1 min and parasites at the same vernier coordinates were tracked and imaged using a light microscope (Eclipse 80i; Nikon, Japan; 100x/1.3 oil immersion lens) fitted with a CCD camera (VB-7010; Keyence, Japan).

### Immunoelectron microscopy (IEM)

Parasite pellets were fixed by incubating in 0.1 M phosphate buffer (pH7.4) containing 4% paraformaldehyde and 0.1% glutaraldehyde on ice for 15 min. The pellets were rinsed thrice with 0.1 M phosphate buffer, dehydrated with ethanol series (30%, 50%, 70%, then 95%), each time at 4°C for 5 min, and embedded in LR White resin (London Resin Company, UK) as described [51]. Thin sections were obtained using an ultramicrotome (Reichert-Jung, Austria) and blocked at 37°C for 30 min with 5% non-fat milk (Becton, Dickinson, and Company, NJ) and 0.0001% Tween 20 (Wako) in PBS (PBS-MT). Sections on the grid were then incubated at 4°C overnight with mouse anti-myc antibody (1:100; 9B11) or control normal mouse IgG in PBS-MT. After washing with PBS containing 5% Blocking One buffer (Nacalai Tesque) and 0.0001% Tween 20 (PBS-BT), the sections were incubated at 37°C for 1 h with goat anti-mouse IgG conjugated to 15 nm gold particles (EY Laboratories, CA) diluted 1:20 in PBS-MT, rinsed with PBS-BT, and fixed with 0.5% OsO_4_ (Nacalai Tesque) at RT for 5 min to stabilize the gold. Finally, the sections were rinsed with distilled water, dried, and stained with uranyl acetate and lead citrate. Samples were examined at 80 kV under a transmission electron microscope (JEM-1230; JEOL Ltd., Japan). The images were processed using Adobe Photoshop CS.

### SDS-PAGE and immunoblotting

Protein fractions were prepared from erythrocytes infected with asynchronous parasites expressing either rPkSBP1 or rPk2TM-a after 0.15% saponin permeabilization in PBS containing a protease inhibitor (PI) cocktail tablet (cOmplete, EDTA-free; Roche Basel, Switzerland) and 1 mM EDTA (PBS-PI). The parasites were pelleted, washed with PBS-PI, and frozen at −80°C until further use. The water soluble fraction (FT) was obtained by freeze-thawing the pellet thrice at −80°C. The parasites were washed thrice with PBS-PI. A 1% solution of Triton X-100 (Tx; Calbiochem, San Diego, CA) detergent was used to resuspend the parasite pellets and the water insoluble fraction was collected after incubation on ice for 30 min. Tx-insoluble pellets were washed thrice with PBS-PI containing Tx. The Tx-insoluble fraction was extracted with 2% sodium dodecyl sulfate (SDS; Nacalai Tesque) in PBS-PI at RT for 30 min. Parasite extracts were adjusted for equal loading (~10^7^/lane) and were subjected to electrophoresis on 5–20% SDS-polyacrylamide gradient mini gels (ATTO, Japan) in a reducing condition. The proteins were transferred onto polyvinylidene fluoride (PVDF) membranes (Merck Millipore) and probed with mouse anti-myc monoclonal antibody (final 1:1000) at RT for 1 h. The blotted membrane was incubated with a solution containing horseradish peroxidase (HRP)-conjugated goat anti-mouse IgG secondary antibody (final 1:25,000, Promega) at RT for 30 min. Mouse monoclonal antibody against *Plasmodium berghei* heat-shock protein 70 (HSP70) that cross-reacts with the orthologs of other *Plasmodium* spp. was obtained from J. Sattabongkot (Mahidol University, Thailand) [52]. Reactivity was visualized with Immobilon^™^ Western Chemiluminescent HRP substrate (Merck Millipore) and detected using a chemiluminescence detection system (LAS-4000EPUVmini; Fujifilm, Japan). The relative molecular sizes of the protein bands were calculated based on reference to the molecular size standards (Precision Plus Protein^™^ Dual Color Standards; Bio-Rad).

## Supporting Information

S1 Fig Sequence alignments of PkSBP1 (A) and Pk2TM-a (B) of *P*. *knowlesi* H-DMU line with the database sequence of H strain. Gray mask indicates predicted transmembrane region by TMHMM2.0. Nucleotides masked with pink or green indicate synonymous or non-synonymous substitutions, respectively. Amino acid residues masked with green indicate amino acid substitutions. Cyan mask indicates indels. Red small letters indicate predicted intron region in *Plasmo*DB. Asterisks indicate positions with identical nucleotides.
(PDF)

S2 Fig Membranous structures in the monkey erythrocytes infected with wild type *P*. *knowlesi* H-DMU line showed no reaction with anti-myc antibody. Slit-like clefts (**A**) and oblong vesicular clefts (**B**) in the erythrocyte cytoplasm were visible in transmission electron micrographs, but were not stained with anti-myc antibody. c, clefts; EM, erythrocyte membrane; P, parasite. Scale bar and sizes are indicated.
(TIF)

S3 Fig No significant difference was detected in the number of anti-myc antibody-positive dots between monkey erthrocyte and human erythrocyte infected with PkSBP1-transgenic *P*. *knowlesi* H_hu_-HSPH line. Statistical difference was examined by Mann-Whitney test (*n* = 30).
(TIF)

S4 Fig Membranous structures in the human erythrocytes infected with wild type *P*. *knowlesi* H_hu_-HSPH line showed no reaction with anti-myc antibody. Slit-like clefts (**A**) and oblong vesicular clefts (**B**) in the erythrocyte cytoplasm were visible in transmission electron micrographs, but not were not stained with anti-myc antibody. c, clefts; EM, erythrocyte membrane; P, parasite. Scale bar and sizes are indicated.
(TIF)

S1 Video 3D reconstructed image of a parasite line expressing rPkSBP1 infecting a human erythrocyte. A representative infected erythrocyte stained with anti-myc antibody (green) for rPkSBP1 and erythrocyte membrane with anti-human CD235a (α-GlyA, red) and showing the cytoplasmic and peripheral distribution of anti-myc antibody-positive puncta. DAPI nucleus-staining (blue).
(MP4)
