# Supplementary material for: Plasmodium knowlesi Skeleton-Binding Protein 1 Localizes to the ‘Sinton and Mulligan’ Stipplings in the Cytoplasm of Monkey and Human Erythrocytes
Source: PLoS One. 2016 Oct 12;11(10):e0164272. doi: 10.1371/journal.pone.0164272 (PMC5061513; doi:10.1371/journal.pone.0164272)
Supplement: S1 Fig — Gray mask indicates predicted transmembrane region by TMHMM2.0. Nucleotides masked with pink or green indicate synonymous or non-synonymous substitutions, respectively. Amino acid residues masked with green indicate amino acid substitutions. Cyan mask indicates indels. Red small letters indicate predicted intron region in PlasmoDB. Asterisks indicate positions with identical nucleotides. (PDF) [file pone.0164272.s001.pdf]

A. PkSBP1

```
                                      S  S  S  H  N  P  S  T  E  E  V  H  H  P  V  P  T  T  V  P  E  V  Q  N  M  G  D  V  N  A  T  A  T
H-DMU                                TCCAGTTCTCACAATCCCAGCACTGAAGAAGTACATCATCCAGTACCCACCACCGTTCCCGAAGTGCAAAATATGGGAGATGTTAATGCTACCGCTACC 99
PKNH_1032400    ATGTGTCAAGCATTTGCAAGTTCCAGTTCTCACAATCCCAGCACTGAAGAAGTACATCATCCAGTACCCACCACCGTTCCCGAAGTGCAAAATATGGGAGATGTTAATGCTACCGCTACC 120
                 M  C  Q  A  F  A  S  S  S  S  H  N  P  S  T  E  E  V  H  H  P  V  P  T  T  V  P  E  V  Q  N  M  G  D  V  N  A  T  A  T
                                     ***************************************************************************************************

                 T  T  T  V  A  T  P  V  P  T  P  V  P  T  P  A  P  A  P  A  P  A  P  V  I  A  T  T  T  E  T  P  S  V  P  D  V  S  T  N
H-DMU           ACTACCACTGTCGCTACCCCTGTCCCTACCCCTGTCCCTACCCCTGCTCCTGCCCCTGCCCCTGCCCCTGTCATTGCCACTACCACTGAAACCCCCTCCGTACCAGATGTATCCACTAAT 219
PKNH_1032400    ACTACCACTGTCACTACCCCTGTCCCTACCCCTGTCGCTACCCCTGCCCCTGCCCCTACCCCTGCCCCTGTCACTACCACTGAAACCACCCCCCCGCCGTACCAGATGTACCCACTAAT 240
                 T  T  T  V  T  T  P  V  P  T  P  V  A  T  P  A  P  A  P  T  P  A  P  V  T  T  T  E  T  T  P  P  A  V  P  D  V  P  T  N
                ************ *********************** ********** ********* *************** * *****   **     ***** ************** ********

                 E  G  A  V  E  I  G  Q  L  S  N  D  V  V  A  D  L  A  S  M  P  D  E  T  A  E  -  H  A  V  A  E  E  P  S  E  V  Q  A  D
H-DMU           GAAGGTGCCGTAGAGATAGGTCAGTTATCTAATGATGTTGTTGCTGACTTGGCCAGCATGCCTGATGAAACCGCTGAA---CATGCAGTTGCTGAGGAACCCTCAGAAGTGCAAGCTGAT 336
PKNH_1032400    GAAGGTGCCGTAGAGATAGGTCAGTTATCTAATGATGTTGTTGCTGACTTGGCCAGCATGCCTGATGAAGCCACTGAACAACATGCAGTTGCTGAGGAACCCTCAGAAGTGCAAGCTGAT 360
                 E  G  A  V  E  I  G  Q  L  S  N  D  V  V  A  D  L  A  S  M  P  D  E  A  T  E  Q  H  A  V  A  E  E  P  S  E  V  Q  A  D
                ********************************************************************* ** *****   ***************************************

                 A  P  V  A  E  S  A  E  E  E  T  I  L  A  P  P  N  L  D  E  M  F  S  E  E  S  I  R  K  L  R  E  S  I  E  N  S  P  C  Y
H-DMU           GCTCCAGTCGCGGAAAGTGCAGAAGAAGAAACCATCTTAGCACCACCTAATCTGGATGAAATGTTCAGTGAAGAATCCATTAGGAAATTGAGGGAATCCATTGAAAACTCCCCATGCTAC 456
PKNH_1032400    GCTCCAGTCGCGGAAAGTGCAGAAGAAGAAACCATCTTAGCACCACCTAATCTGGATGAAATGTTCAGTGAAGAATCCATTAGGAAATTGAGGGAATCCATTGAAAACTCCCCATGCTAC 480
                 A  P  V  A  E  S  A  E  E  E  T  I  L  A  P  P  N  L  D  E  M  F  S  E  E  S  I  R  K  L  R  E  S  I  E  N  S  P  C  Y
                ************************************************************************************************************************

                 Q  R  R  L  A  A  Y  R  E  Q  Q  E  R  L  G  K  S  I  D  I  S  P  S  S  P  M  I  S  P  L  Y  K  L  Q  F  F  G  S  Y  A
H-DMU           CAACGTAGATTAGCTGCCTATAGAGAACAACAAGAAAGACTTGGAAAATCTATCGACATCAGCCCATCCTCACCTATGATATCTCCATTATACAAGTTACAATTCTTCGGAAGCTATGCC 576
PKNH_1032400    CAACGTAGATTAGCTGCCTATAGAGAACAACAAGAAAGACTTGGAAAATCTATCGACATCAGCCCATCCTCACCTATGATATCTCCATTATACAAGTTACAATTCTTCGGAAGCTATGCC 600
                 Q  R  R  L  A  A  Y  R  E  Q  Q  E  R  L  G  K  S  I  D  I  S  P  S  S  P  M  I  S  P  L  Y  K  L  Q  F  F  G  S  Y  A
                ************************************************************************************************************************
```

```
                     K  G  M  I  Q  L  I  Q  K  N  Y  L  M  V  L  L  I  G  L  F  I  M  N  G  I  L  F  Y  N  Y  Y  K  G  S  A  G  K  K  K  C
H-DMU               AAGGGGATGATTCAATTAATACAAAAGAACTACTTAATGGTTTTACTCATTGGATTGTTCATAATGAACGGTATCCTGTTCTATAATTACTATAAAGGTTCCGCAGGCAAGAAAAAATGC 696
PKNH_1032400        AAGGGGATGATTCAATTAATACAAAAGAACTACTTAATGGTTTTACTCATTGGATTGTTCATAATGAACGGTATCCTGTTCTATAATTACTATAAAGGTTCCGCAGGCAAGAAAAAATGC 720
                     K  G  M  I  Q  L  I  Q  K  N  Y  L  M  V  L  L  I  G  L  F  I  M  N  G  I  L  F  Y  N  Y  Y  K  G  S  A  G  K  K  K  C
                    ************************************************************************************************************************

                     K  E  E  K  L  K  K  K  M  K  A  K  C  K
H-DMU               AAGGAAGAAAAACTCAAAAAAAAAATGAAAGCAAAATGTAAGA
PKNH_1032400        AAGGAAGAAAAACTCAAAAAAAAAATGAAAGCAAAATGTAAGAAACTCGATGGAGATGCCATTTAA 786
                     K  E  E  K  L  K  K  K  M  K  A  K  C  K  K  L  D  G  D  A  I
                    *******************************************
```

B. Pk2TM-a

```
                                          P  L  I  K  I  L  T  F  S  A  L  V  W  I  S  P  N  S  Y  R
H-DMU                                    CCTTTAATTAAAATTCTTACATTTAGTGCCTTAGTATGGATATCTCCCAACTCTTATAGGgtacagtaattaattatggactaatagtttagatacttc
PKNH_0623600        ATGAAAGAAATATTTCTGTTCCCTTTAATTAAAATTCTTACATTTAGTGCCTTAGTATGGATATCTCCCAACTCTTATAGGgtacagtaattaattatggactaatagtttagatacttc 120
                     M  K  E  I  F  L  F  P  L  I  K  I  L  T  F  S  A  L  V  W  I  S  P  N  S  Y  R
                                         ***************************************************************************************************

H-DMU               gctgtccatttattatgggcaatttaagaatattcttcattttattaatgtgaaaataagtgaacaagaatgaattttgttttacacattttatgtaactttttttgtttgtctttcgtgc
PKNH_0623600        gctgtccatttattatgggcaatttaagaatattcttcattttattaatgtgaaaataagtgaacaagaatgaattttgttttacacattttatgtaactttttttgtttgtctttcgtgc 240
                    ************************************************************************************************************************

                       S  A  I  S  G  E  L  W  E  N  R  I  G  L  Y  D  K  L  E  L  R  N  S  R  L  L  S  E  R  W  K  Q  Y  R  H  E  R  N  V
H-DMU               agTCAGCTATTTCGGGGGAATTATGGGAGAACAGAATAGGTTTATATGACAAGTTAGAGCTAAGGAATAGCAGGTTATTAAGTGAAAGGTGGAAGCAATATAGACATGAAAGAAATGTTA
PKNH_0623600        agTCAGCTATTTCGGGGGAATTATGGGAGAACAGAATAGGTTTATATGACAAGTTAGAGCTAAGGAATAGCAGGTTATTAAGTGAAAGGTGGAAGCAATATAGACATGAAAGAAATGTTA 360
                       S  A  I  S  G  E  L  W  E  N  R  I  G  L  Y  D  K  L  E  L  R  N  S  R  L  L  S  E  R  W  K  Q  Y  R  H  E  R  N  V
                    ************************************************************************************************************************
```

```
                 N  L  K  E  R  K  H  N  M  L  K  D  E  D  H  Y  D  E  R  F  P  S  S  M  R  N  K  P  N  P  R  E  H  M  D  N  P  G  F  S
H-DMU           ACTTAAAAGAAAGAAAACATAATATGCTAAAAGATGAGGATCATTATGACGAAAGATTCCCAAGTAGTATGAGGAATAAACCTAATCCTCGGGAACACATGGATAATCCAGGATTTAGTG
PKNH_0623600    ACTTAAAAGAAAGAAAACATAATATGCTAAAAGATGAGGATCATTATGACGAAAGATTCCCAAGTAGTATGAGGAATAAACCTAATCCTCGGGAACACATGGATAATCCAGGATTTAGTG 480
                 N  L  K  E  R  K  H  N  M  L  K  D  E  D  H  Y  D  E  R  F  P  S  S  M  R  N  K  P  N  P  R  E  H  M  D  N  P  G  F  S
                ************************************************************************************************************************

                 E  K  T  D  K  L  T  S  R  S  K  Y  G  N  H  F  G  R  R  K  D  K  I  A  N  E  K  I  H  R  T  S  K  G  N  S  E  I  D  E
H-DMU           AGAAAACTGATAAATTAACATCCAGAAGTAAATATGGTAATCATTTTGGAAGAAGAAAAGATAAAATAGCAAATGAAAAAATTCATAGAACTTCAAAGGGGAATTCTGAAATAGATGAAG
PKNH_0623600    AGAAAACTGATAAATTAACATCCAGAAGTAAATATGGTAATCATTTTGGAAGAAGAAAAGATAAAATAGCAAATGAAAAAATTCATAGAACTTCAAAGGGGAATTCTGAAATAGATGAAG 600
                 E  K  T  D  K  L  T  S  R  S  K  Y  G  N  H  F  G  R  R  K  D  K  I  A  N  E  K  I  H  R  T  S  K  G  N  S  E  I  D  E
                ************************************************************************************************************************

                 D  D  N  Y  L  K  E  P  E  D  G  Y  Y  M  R  E  G  N  V  E  Y  G  G  G  N  K  E  Y  K  K  P  P  N  N  N  V  H  G  I  Q
H-DMU           ATGATAATTATCTTAAAGAGCCGGAGGACGGTTACTATATGAGAGAAGGAAATGTTGAATATGGAGGAGGAAATAAAGAATATAAAAAACCACCAAATAATAATGTTCATGGAATACAGA
PKNH_0623600    ATGATAATTATCTTAAAGAGCCGGAGGACGGTTACTATATGAGAGAAGGAAATGTTGAATATGGAGGAGGAAATAAAGAACATAAAAAACCACCAAATAATAATGTTCATGGAATACAGA 720
                 D  D  N  Y  L  K  E  P  E  D  G  Y  Y  M  R  E  G  N  V  E  Y  G  G  G  N  K  E  H  K  K  P  P  N  N  N  V  H  G  I  Q
                ******************************************************************************** ***************************************

                 S  K  S  G  M  N  N  K  N  L  E  K  L  P  R  P  P  N  E  N  V  P  Y  T  L  E  Y  Y  H  Y  S  K  V  K  R  G  V  M  E  K
H-DMU           GTAAATCCGGAATGAATAATAAAAATTTGGAGAAACTTCCCCGACCACCAAATGAGAACGTTCCTTACACATTGGAATACTACCATTATAGCAAAGTAAAGAGAGGTGTAATGGAGAAGT
PKNH_0623600    GTAAATCCGGAATGAATAATAAAAATTTGGAGAAACTTCCCCGACCACCAAATGAGAACGTTCCTTACACATTGGAATACTACCATTATAGCAAAGTAAAGAGAGGTGTAATGGAGAAGT 840
                 S  K  S  G  M  N  N  K  N  L  E  K  L  P  R  P  P  N  E  N  V  P  Y  T  L  E  Y  Y  H  Y  S  K  V  K  R  G  V  M  E  K
                ************************************************************************************************************************

                 F  V  N  A  L  K  K  M  D  V  Q  F  Q  L  K  F  M  R  Y  M  K  A  R  N  Y  S  A  E  R  E  F  L  E  L  R  S  R  K  E  I
H-DMU           TCGTAAATGCACTTAAAAAAATGGATGTACAATTTCAATTAAAATTTATGCGTTACATGAAAGCGAGGAATTATTCGGCGGAACGTGAATTTTTGGAACTTCGAAGTAGGAAAGAAATAA
PKNH_0623600    TCGTAAATGCACTTAAAAAAATGGATGTACAATTTCAATTAAAATTTATGCGTTACATGAAAGCGAGGAATTATTCTGCGGAACGTGAATTTTTGGAACTTCGAAGTAGGAAAGAAGTAA 960
                 F  V  N  A  L  K  K  M  D  V  Q  F  Q  L  K  F  M  R  Y  M  K  A  R  N  Y  S  A  E  R  E  F  L  E  L  R  S  R  K  E  V
                ***************************************************************************** *************************************** ***
```

```
              I  S  Y  Y  F  R  R  Y  K  I  M  L  P  L  I  M  Q  T  V  I  F  S  I  F  L  G  L  T  L  T  T  S  A  V  G  P  M  F  I  M
H-DMU         TTTCTTATTATTTTAGGAGGTACAAAATAATGTTGCCACTTATAATGCAAACTGTCATTTTTTCGATATTCTTAGGGTTGACACTTACAACTAGTGCAGTCGGTCCAATGTTCATAATGT
PKNH_0623600  TTTCTTATTATTTTAGGAGGTACAAAATAATGTTGCCACTTATAATGCAAACTGTCATTTTTTCGATATTCTTAGGGTTGACACTTACAACTAGTGCAGTCGGTCCAATGTTCATAATGT 1080
              I  S  Y  Y  F  R  R  Y  K  I  M  L  P  L  I  M  Q  T  V  I  F  S  I  F  L  G  L  T  L  T  T  S  A  V  G  P  M  F  I  M
              ************************************************************************************************************************

              S  C  I  T  G  E  L  L  L  N  L  S  Y  Y  Y  L  K  A  Y  I  R  I  K  K  I  H  R  N  F  R  Q
H-DMU         CTTGTATTACGGGTGAATTGCTCTTGAATTTGTCTTACTATTATTTAAAAGCATATATTAGAATAAAAAAAATTCACAGAAATTTCAGACAGC
PKNH_0623600  CTTGTATTACGGGTGAATTGCTCTTGAATTTGTCTTACTATTATTTAAAAGCATATATTAGAATAAGAAAAATTCACAGAAATTTCAGACAGCACGGTGTTAAAATTAAAGAATGA 1196
              S  C  I  T  G  E  L  L  L  N  L  S  Y  Y  Y  L  K  A  Y  I  R  I  R  K  I  H  R  N  F  R  Q  H  G  V  K  I  K  E
              ******************************************************************* *************************
```

Fig. S1. Sequence alignments of PkSBP1 and Pk2TM-a of *Plasmodium knowlesi* H-DMU line with the database sequence of H strain. Gray mask indicates predicted transmembrane region by TMHMM2.0. Nucleotides masked with pink or green indicate synonymous or non-synonymous substitutions, respectively. Amino acid residues masked with green indicate amino acid substitutions. Cyan mask indicates indels. Red small letters indicate predicted intron region in *Plasmo*DB. Asterisks indicate positions with identical nucleotides.
