# Supplementary figures and images for: Plasmodium knowlesi Skeleton-Binding Protein 1 Localizes to the ‘Sinton and Mulligan’ Stipplings in the Cytoplasm of Monkey and Human Erythrocytes
Source: PLoS One. 2016 Oct 12;11(10):e0164272. doi: 10.1371/journal.pone.0164272 (PMC5061513; doi:10.1371/journal.pone.0164272)

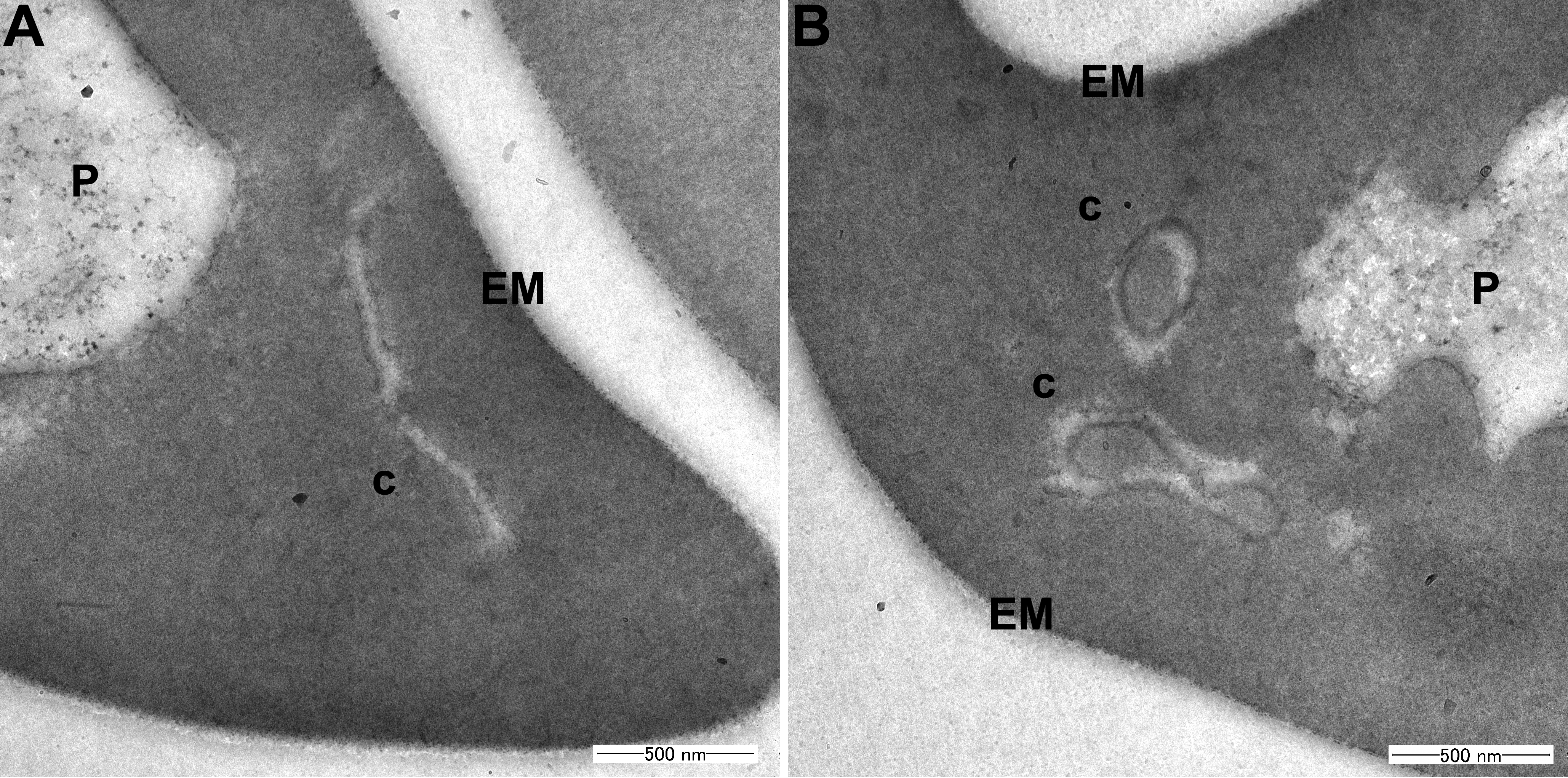

Supplement: S2 Fig — Slit-like clefts (A) and oblong vesicular clefts (B) in the erythrocyte cytoplasm were visible in transmission electron micrographs, but were not stained with anti-myc antibody. c, clefts; EM, erythrocyte membrane; P, parasite. Scale bar and sizes are indicated. (TIF) [file pone.0164272.s002.tif]

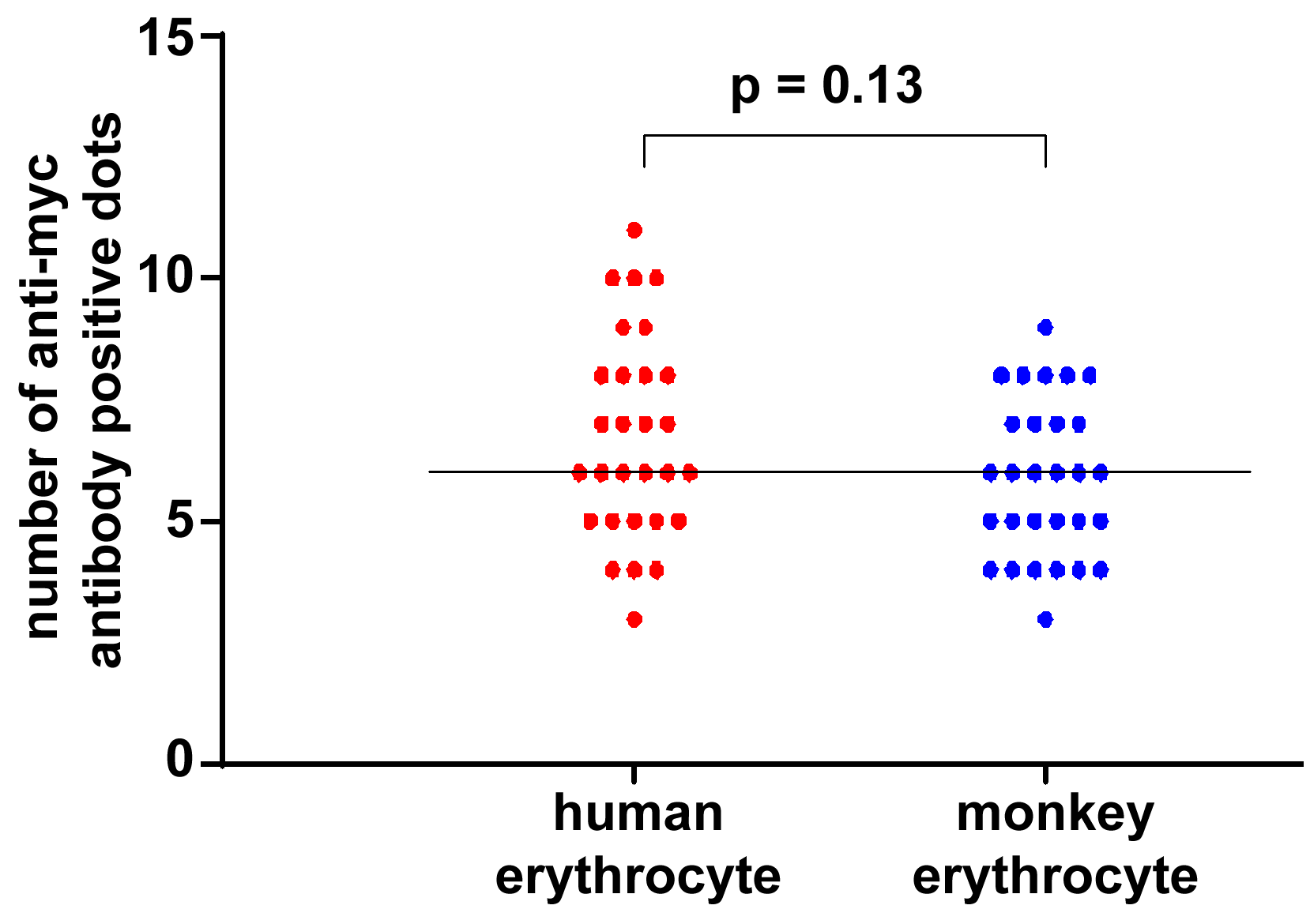

Supplement: S3 Fig — Statistical difference was examined by Mann-Whitney test (n = 30). (TIF) [file pone.0164272.s003.tif]

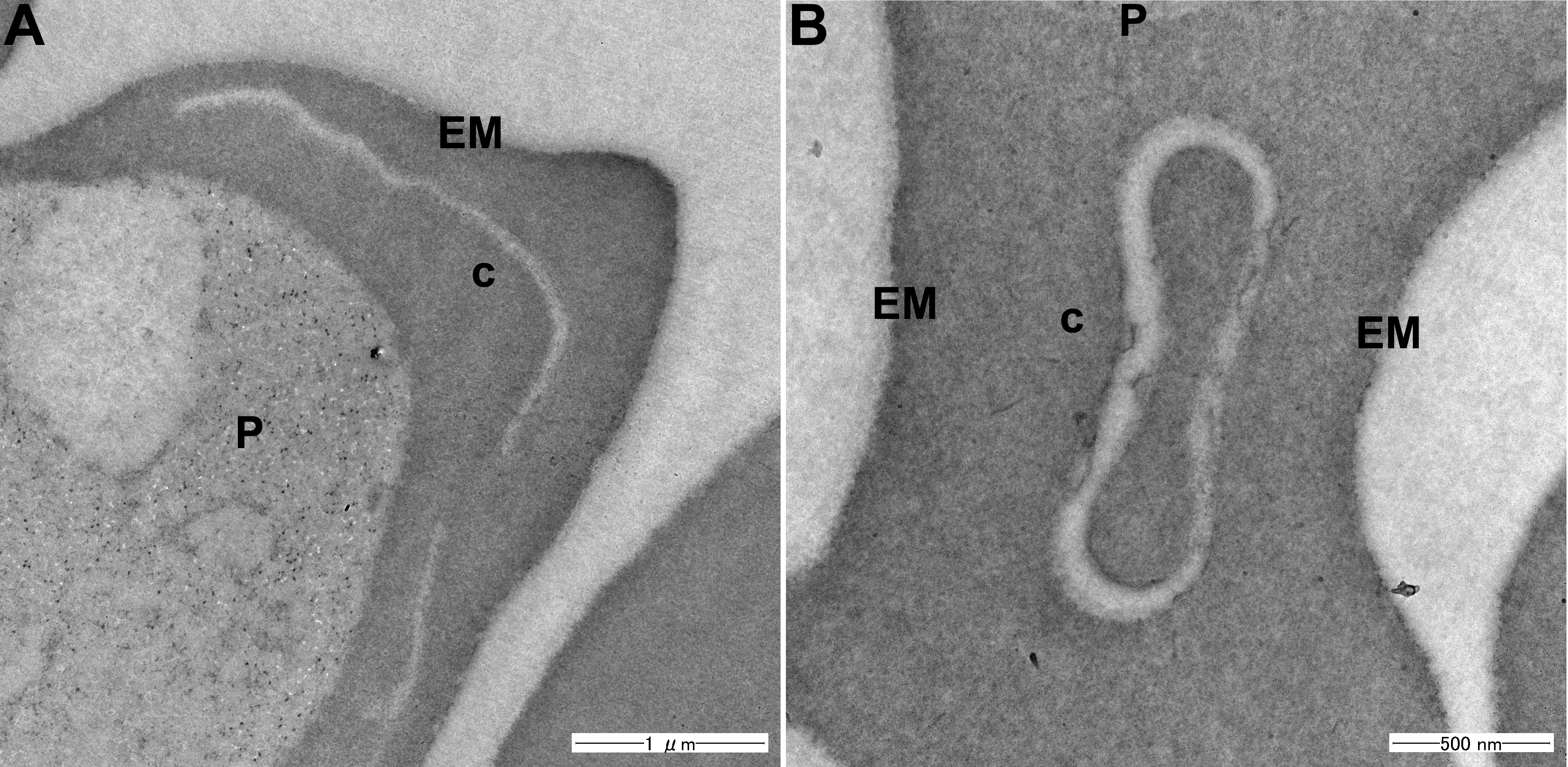

Supplement: S4 Fig — Slit-like clefts (A) and oblong vesicular clefts (B) in the erythrocyte cytoplasm were visible in transmission electron micrographs, but not were not stained with anti-myc antibody. c, clefts; EM, erythrocyte membrane; P, parasite. Scale bar and sizes are indicated. (TIF) [file pone.0164272.s004.tif]
